# Consumer neuroscience to inform consumers—physiological methods to identify attitude formation related to over-consumption and environmental damage

**DOI:** 10.3389/fnhum.2014.00304

**Published:** 2014-05-20

**Authors:** Peter Walla, Monika Koller, Julia L. Meier

**Affiliations:** ^1^Functional Neuroimaging Lab, Centre for Translational Neuroscience and Mental Health Research, School of Psychology, University of NewcastleNewcastle, NSW, Australia; ^2^Department of Strategic Management, Marketing and Tourism, University of Innsbruck School of ManagementInnsbruck, Austria; ^3^Department of Marketing, Vienna University of Economics and BusinessVienna, Austria

**Keywords:** consumer neuroscience, emotion, attitude, objective measures, subjective measures, startle reflex modulation

## Introduction

Climate change, the need for efficient and environment-friendly energy use and health-related issues like obesity and addictions, these three crucial topics build a triad that the global society has extensively been discussing and caring about during the past decades. First, according to the recently published fifth IPCC climate change assessment report (2013), intense weather conditions have been on the rise. These changes will in extreme cases impose life-threatening dangers to some civilizations, but it will mostly influence individual attitudes and decision-making and thus finally modify consumption behavior quite dramatically during the next decades. Second, the European Union is aiming for a 20% cut in Europe's annual primary energy consumption by 2020 (Energy Efficiency Plan, 2011). This government-driven aim does not only affect global industry, but again also the consumption behavior of each individual end-user. Third, according to the World Health Organization (2013), worldwide obesity has nearly doubled since 1980. In fact, 65% of the world's population lives in countries where overweight and obesity kills more people than underweight (World Health Organization, 2013). Given these unpleasant scenarios we need to get active now in order to prevent the worst and to ensure the best possible and highest standards of life across the globe.

## Targeted research is requested

Fortunately, Horizon 2020, the seventh framework program of the European Commission relates to the abovementioned areas as being important research topics, which need to be investigated more comprehensively until 2020 (European Commission, 2013). However, given their rather global political nature how can reliable respective research be done ideally taking all of these three main concerns into account in one go while at the same time providing useful insight?

Decision-making and attitude formation seem to be the common denominator. What are the true attitudes of an average consumer as the smallest unit in a society? What does a consumer think about his/her individual role within these current dynamics? After all, the actual goal is to understand why one is over-consuming, pursuing an unhealthy lifestyle and wasting household energy, although actually knowing about the negative consequences? Unfortunately, knowing the right questions is not enough, we also need adequate research that provides reliable answers, which let us understand and predict human behavior, particularly consumers' choices. Of course, a lot of research has been done, but traditional research approaches are potentially misleading as highlighted by various studies comparing explicit with implicit responses (Spector, 1994).

## What alternative measures do we have?

Ultimately, all human behavior is a consequence of both cognitive and affective processing, but only cognitive aspects can be reliably captured through questionnaire-based investigations, while affective processing happens largely outside our awareness (Walla and Panksepp, 2013). Consumer decision-making and attitude formation are strongly engaging affective processing and incomplete understanding and suboptimal investigations might come along with severely negative consequences for the individual as well as for the society and the entire environment. Just to think of one example, when it comes to study the acceptance of new household technologies that are more energy efficient one could get strongly biased and misleading results if only using self-report approaches. Consequently, the potential gap between explicitly stated intention to use power saving household tools and actual future behavior would not help to improve the situation. Especially, topics related to environmental issues are prone to be biased by social desirability and various other often unknown pressures (Glasman and Albarracin, 2006). Similarly, issues related to eating behavior resulting in overweight do rarely have pure cognitive origins, but most commonly are based on emotions grounding on an affectionate and attitude based source. Among these are emotion regulation, fear and stress (Kemp et al., 2013).

Given this major shortcoming of self-reports in terms of measuring affective processing in the consumer brain, neuroscientific methods are here suggested to provide added value in this respect. However, not all methods from consumer neuroscience are equally suitable. Besides its merits to gather information on fundamental experimental effects, the capabilities of brain imaging techniques like fMRI (functional Magnet Resonance Imaging) to study real consumption behavior are limited. In case one wants to study purchase decisions directly at the point of sale the use of fMRI renders itself impossible. In addition, fMRI is a very expensive technology. Luckily, more affordable while also more reliable methods are available to make the desired progress in studying consumer likes and dislikes, their attitudes and finally to predict their actual behavior. Recent studies that focused on comparisons between explicit and implicit measures of affect as in like and dislike clearly showed that discrepancies between self-reported like and objectively measured like can occur (Geiser and Walla, 2011; Grahl et al., 2012; Mavratzakis et al., 2013). From these and a number of other studies it can be concluded that whenever such discrepancies occur respective self-reported likes or dislikes have to be treated with great caution.

Some of these studies utilized a long known approach to tap into non-conscious raw affective processing in the human brain, a phenomenon known as startle reflex modulation (SRM). SRM is a valid approach to selectively measure the valence of affective information processing. It is easy to apply also in a field setting and can perfectly be combined with methods measuring the associated level of arousal, like skin conductance and heart rate (Dawson et al., 1999; Walla et al., 2011). Measuring the underlying forces of behavior other than mere stated intention is also crucial regarding health-related issues with respect to consumption. Applying neuroscientific methods like SRM and maybe electroencephalography (EEG) (Bosshard and Walla, 2013) can be useful in this regard as well (e.g., Walla et al., 2010 used these methods to study the emotion impact of food intake).

## What specific research should we focus on?

Although obviously different in many respects, a closer look at climate change, environment-friendly energy as well as health reveals that their individually associated behaviors are all remarkably dependent on attitudes. Attitudes form important bases for behavior and thus knowing them allows us to predict behavior. For instance, an attitude like “global warming is natural, not man-made” is likely associated with future behavior that does not really do anything against it. Also, health issues do of course strongly depend on the right attitudes. Attitudes are consciously (explicitly) and non-consciously (implicitly) learned and as such are prone to change be it driven by more or less random individual experiences or by planned and strategic political or marketing campaigns. They are shaped by intellectual elaboration, but they also have a strong affective component, which reflects aspects of basic like or dislike. Crucially, and that is what makes attitudes empirically testable, they can be modified as a consequence of learning processes such as conditioning (e.g., Hofmann et al., 2010). In particular, evaluative conditioning is often used where for instance “nature” is repeatedly paired with positive unconditioned stimuli to finally create a positive nature-attitude in children preparing them for a nature protecting adulthood. Through evaluative conditioning, *unknown* and even *disliked* can be turned into *liked*. Obviously, evaluative conditioning and its effects on attitudes seems to be a very promising and meaningful field to investigate. Together with neuroscientific methods and techniques the investigation of attitudes, their formation and their changes are here clearly emphasized as being most successful when it comes to predicting human behavior (see Bosshard and Walla, 2013).

## The main take home message

Crucially, and this forms the main take home message of this opinion article, the use of surveys only taps into the explicit (conscious) aspect of a consumer's attitude, whereas implicit aspects are not at all reflected in self-reported data (see Rugg et al., 1998; Walla et al., 1999). This is potentially misleading, because implicit attitudes have been shown to be better predictors of particularly spontaneous behavior (Gawronski and Bodenhausen, 2012). Explicit attitudes are deliberate evaluations formulated through reasoning and consequently even if the individual subjectively perceives their outlook toward it to be positive, negative associations can be activated, and vice versa (Devine, 1989). Implicit attitudes are independent of higher cognitive resources and occur irrespective of their alignment with the individual's introspective assessment. They are non-conscious and thus only accessible via objective measures such as SRM and EEG.

## Talk between science community and society

It is necessary to continuously engage in an educated dialog with the average consumer. This means that one precondition for being able to realize such a dialog is to translate the research findings into a language that is actually being understood by the society. Doing so allows creating added value for the society through science. In a nutshell, this opinion article outlines how consumer neuroscience may be used to create societal value. The more we know about non-conscious processes that drive human behavior the more each individual consumer knows and thus can better understand and finally control his own behavior. Neuroscientific methods in general and SRM and EEG in particular, might serve as valid instruments for addressing consumption-related issues of the topical triad that are important to build a larger picture in terms of society and well-being. This opinion article may provide vital insights for advancing academic knowledge but also provide the basis for guidelines for experts and policy makers.

The authors of this article declare that the research was conducted in the absence of any commercial or financial relationships that could be construed as a potential conflict of interest. At no time did the authors or their institutions receive payment or services from a third party for any aspect of the submitted work.

## Author contributions

All three authors (Peter Walla, Monika Koller, and Julia L. Meier) meet all of the below listed criteria:

- Substantial contributions to the conception or design of the work; or the acquisition, analysis, or interpretation of data for the work; AND
- Drafting the work or revising it critically for important intellectual content; AND
- Final approval of the version to be published; AND
- Agreement to be accountable for all aspects of the work in ensuring that questions related to the accuracy or integrity of any part of the work are appropriately investigated and resolved.

### Conflict of interest statement

The Associate Editor, Dr. Nick Lee declares that, despite having collaborated with the author Dr. Monika Koller, the review process was handled objectively and no conflict of interest exists. The authors declare that the research was conducted in the absence of any commercial or financial relationships that could be construed as a potential conflict of interest.
